# Advancing brain health equity with Indigenous peoples: A critical imperative

**DOI:** 10.1002/alz.71125

**Published:** 2026-02-25

**Authors:** Antonia J. Clarke, Cliff Whetung, Astrid Suchy‐Dicey, Adrienne Withall, Kylie Radford, Diane C. Gooding, Louise Lavrencic, Makarena Dudley, Dina Lo Giudice, Leon Flicker, Arantxa Sanchez Boluarte, Sulakshna Aggarwal, Kyle R. Conniff, Amy G. Brodtmann, Monica M. Diaz, Stéfanie A. Tremblay, Emmanuel S. Nwofe, Carey E. Gleason, Kristen Jacklin, Joseph Keawe'aimoku Kaholokula, Chontel Gibson, Juliana Souza‐Talarico, Pamela Roach, the Alzheimer's Association Intermission 2025: Stronger Together Collaboration

**Affiliations:** ^1^ School of Translational Medicine Monash University Melbourne Victoria Australia; ^2^ Department of Family Medicine and Biobehavioural Health Memory Keepers Medical Discovery Team University of Minnesota Minneapolis Minnesota USA; ^3^ Slone Epidemiology Center Chobanian & Avedisian School of Medicine Boston University Boston Massachusetts USA; ^4^ School of Psychology University of New South Wales Sydney New South Wales Australia; ^5^ School of Psychology University of New South Wales Kensington New South Wales Australia; ^6^ Wisconsin Alzheimer's Disease Research Center University of Wisconsin–Madison School of Letters and Sciences University of Wisconsin–Madison School of Medicine and Public Health Madison Wisconsin USA; ^7^ Neuroscience Research Australia University of New South Wales Kensington New South Wales Australia; ^8^ School of Psychology Centre for Brain Research University of Auckland Auckland New Zealand; ^9^ Department of Medicine Dentistry and Health Sciences University of Melbourne Melbourne Victoria Australia; ^10^ Medical School University of Western Australia Perth Western Australia Australia; ^11^ Center for Global Health Universidad Peruana Cayetano Heredia Heredia Peru; ^12^ Maulana Azad Medical College New Delhi India; ^13^ University of Wisconsin–Madison School of Medicine and Public Health University of Wisconsin–Madison School of Letters and Sciences Madison Wisconsin USA; ^14^ Department of Neurology University of North Carolina at Chapel Hill School of Medicine Chapel Hill North Carolina USA; ^15^ Department of Neurology and Neurosurgery The Neuro McGill University Montreal Quebec Canada; ^16^ Centre for Applied Dementia Studies University of Bradford Bradford UK; ^17^ Wisconsin Alzheimer's Disease Research Center Madison Veteran's Hospital GRECC University of Wisconsin–Madison School of Medicine and Public Health Madison Wisconsin USA; ^18^ Department of Family Medicine and Biobehavioural Health University of Minnesota Minneapolis Minnesota USA; ^19^ John A. Burns School of Medicine University of Hawai'i at Mānoa Honolulu Hawai'i; ^20^ Aboriginal Health and Ageing Team Neuroscience Research Australia Sydney NSW Australia; ^21^ College of Nursing University of Iowa Iowa city USA; ^22^ Departments of Family Medicine and Community Health Services Cumming School of Medicine University of Calgary Calgary Alberta Canada

**Keywords:** Alzheimer's disease and related dementias, brain health, culturally safe care, decolonizing health research, dementia, epidemiology, First Nations, health equity, Indigenous peoples, Indigenous research methodologies, neurodegeneration, social determinants of health

## Abstract

Indigenous communities hold rich knowledges, cultural practices, and kinship networks that sustain cognitive resilience and nurture brain health across the life course. Yet these enduring strengths are often obscured by biomedical models that overlook the structural determinants of health—including the cumulative effects of colonization, educational inequity, and socioeconomic disadvantage—that diminish brain health. Accordingly, dementia disproportionately affects Indigenous populations globally, often presenting with earlier onset and higher prevalence compared to non‐Indigenous groups. This perspective synthesizes the current epidemiological evidence, situates dementia risk within its structural and historical context, and explores how Indigenous knowledges, cultural continuity, and community leadership can inform dementia research and care more broadly. Our global insights call for a decisive shift: to decolonize dementia research and policy and move beyond deficit‐based narratives toward approaches that prioritize Indigenous leadership and culturally grounded, strengths‐based pathways to brain health equity.

**Highlights:**

- This perspective synthesizes global evidence on dementia epidemiology among Indigenous populations, examining structural determinants and Indigenous perspectives on brain health and dementia care.
- Structural inequities and the enduring legacies of colonization, rather than biology alone, underpin the disproportionate dementia burden among Indigenous peoples worldwide.
- Centering culture, kinship, and connection to land and community reframes brain health beyond biomedical models and reveals cultural resilience as a powerful neuroprotective resource.
- Key recommendations call for Indigenous leadership and locally tailored, culturally grounded approaches to advance lifelong brain health equity and develop strengths‐based models of dementia care.

## 1 INTRODUCTION

Dementia is increasingly recognized as a condition shaped across the life course, with its onset reflecting the accumulated imprint of socio‐economic, political, and biological forces. Cognitive resilience, the brain's capacity to adapt and thrive in the face of challenges, is also forged within these contexts.^1^ For Indigenous communities worldwide, cognitive resilience is deeply socio‐cultural, grounded in inter‐generational knowledge systems, land‐based practices, and the strength of community and kinship.^2^, ^3^ These tenets are believed to protect brain health and buffer the enduring impacts of colonization, yet they are often overlooked in dementia research and policy.^4^ Their omission contributes to a disproportionate burden of dementia for Indigenous peoples globally: in high‐income countries (HICs), dementia prevalence is reportedly up to threefold higher than the referent non‐Indigenous populations,^5^, ^6^ with onset occurring a decade or more earlier.^7^, ^8^ Evidence from low‐ and middle‐income countries (LMICs) also reveals marked variation shaped by geography and culture.^9^, ^10^, ^11^ The available data highlight both unique protective and risk factors, the consistent influence of structural inequities, and the cumulative effects of adverse social determinants of health. Advancing brain health equity for Indigenous peoples therefore demands more than biomedical risk reduction. It requires an approach rooted in dismantling systemic barriers to good health and recognizing that dementia risk is context specific and culturally situated. Indigenous leadership is an essential first step to integrating culturally grounded, holistic pathways of resilience into dementia prevention and care.

**Box 1: Definitions of the key constructs identified within Indigenous health and well‐being frameworks, adapted from definitions provided by Chen et al., 2022,^12^ Joshi et al., 2022,^13^ and Udeh‐Momoh et al., 2025.^1^**

**Brain health**: A lifelong continuum of cognitive, socio‐emotional, and spiritual well‐being determined by intergenerational, ecological, psychological, social, and cultural influences.
**Cognitive reserve**: A latent capacity built through lifelong and culturally grounded learning that enables compensation for pathology via flexible neural network recruitment.
**Cognitive resilience**: The capacity to sustain cognitive functioning in the face of structural, social, historical, and health‐related stressors, supported by cultural continuity, kinship, and community strengths.

In this article, “Indigenous” refers to the first peoples of lands colonized predominantly by European powers— communities whose cultural, spiritual, and political identities are rooted in pre‐colonial histories and who endure despite centuries of dispossession and systemic marginalization. Globally, relationships between non‐Indigenous populations and Indigenous peoples in colonized countries remain shaped by colonial ideologies and dominant Western worldviews, sustaining historical and geopolitical inequities in health and well‐being. Yet Indigenous peoples are not passive in colonial contexts, and most, if not all, have never ceded sovereignty to lands, bodies, knowledge systems, culture, spirituality, and spirit. Indigenous people are reclaiming and restoring Indigenous knowledges to strengthen self‐determination and Indigenous human rights. These efforts support the well‐being of Elders and older people, communities, future generations, and a more responsive approach to dementia research, prevention, and care.^3^

**FIGURE 1:**
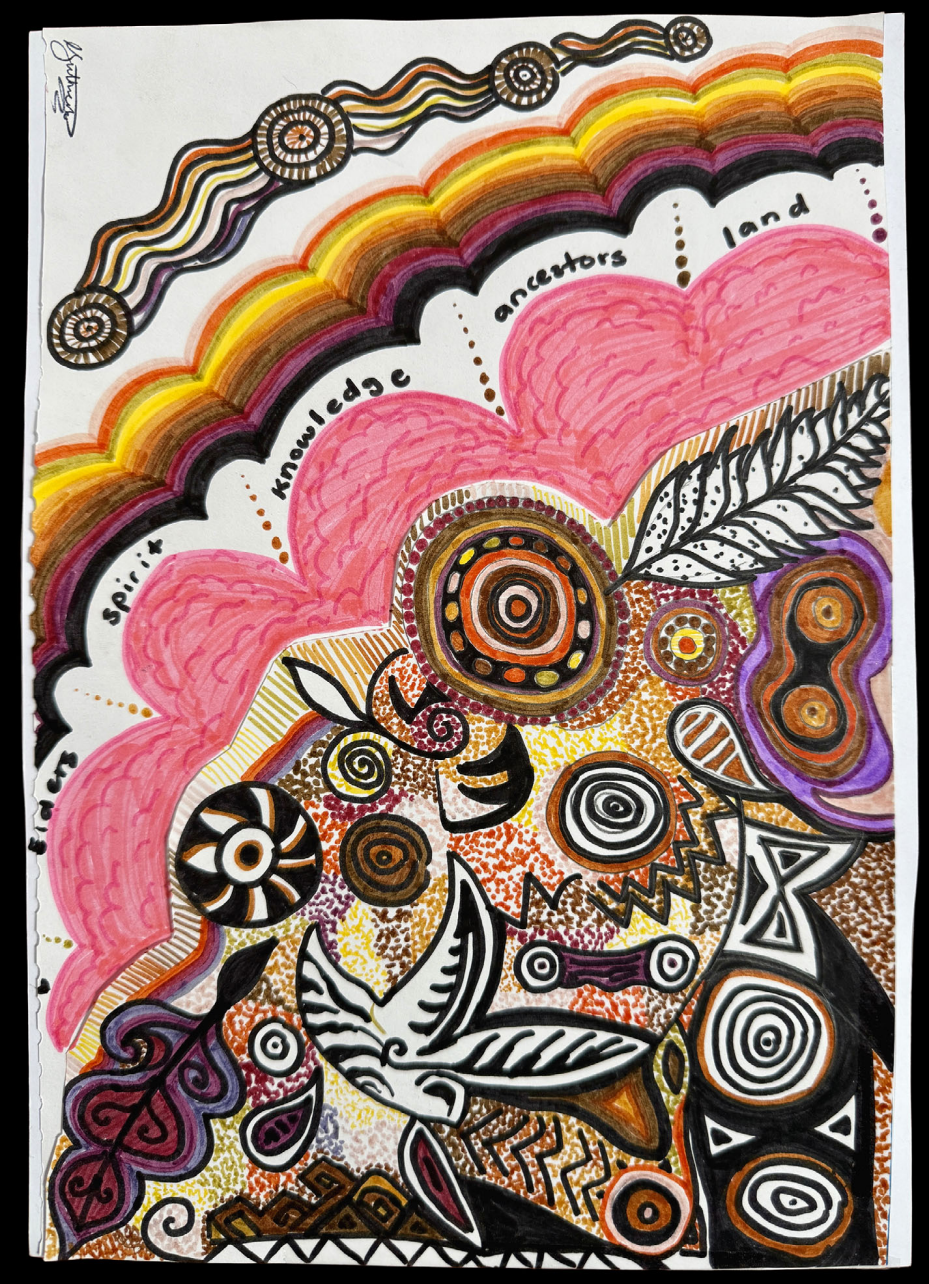
The coming together of many stories and many peoples, across many lands, unified for brain health equity with Indigenous peoples and for the next generation. This is an original artwork commissioned for this manuscript, created by Isla Sutherland, daughter of Associate Professor Adrienne Withall. The artwork incorporates motifs from Aboriginal and Torres Strait Islander, Māori, Canadian First Nations, Inuit, and Métis, American Indian and Alaska Native, Native African, and Native Hawaiian cultures, representing the diversity of voices and ideas that contributed to this manuscript. The curved linear imagery is reminiscent of the folds of the brain, made vibrant by upholding the tenets of Indigenous determinants of health—respect for Elders, cultural knowledge and spirituality, intergenerational connection to ancestors, and responsibility to care for Country. By combining several artistic aspects of each culture, every instroke is a wave of unity, integration, and cultural pride. The rich, earthy colors collide to create a work that is not only connected to people, but to land and all other Indigenous aspects. © 2025 Isla Sutherland. Reproduced with permission.

This review represents the first global collaboration between Indigenous and non‐Indigenous researchers, educators, and clinicians to delineate the methodological and system‐level changes required to advance brain health equity with Indigenous populations (Figure 1). This perspectives piece arises from a collaboration at the 2025 Alzheimer's Association International Conference in Toronto, Canada. We demonstrate that prevailing epidemiological paradigms insufficiently account for the historical, geographical, and structural conditions that shape dementia risk, constraining both evidence and intervention design. By foregrounding Indigenous worldviews, rooted in relationality and holism, we identify conceptual pathways that could strengthen dementia prevention and care policies and advance lifelong brain health more broadly. Box 1 operationalizes this shift by situating the constructs of brain health, cognitive resilience, and cognitive reserve within Indigenous conceptualizations of health.

To support transparency and reflexivity, we present positionality statements from all contributing authors, outlining our cultural, professional, and geographic perspectives in relation to Indigenous knowledges and experiences (Table 1).

**TABLE 1 alz71125-tbl-0001:** The positionality and perspectives offered by all co‐authors.

Name	Positionality
**Antonia Jean Clarke**	*Antonia is a non‐Indigenous neurologist and researcher, born and raised in Aotearoa New Zealand. She trained as a doctor across rural and remote Australia following a previous career in the law and holds a deep interest in health equity and lifelong brain health. She is also a mother of three children. These personal and professional experiences inform her appreciation of Aboriginal well‐being, which is deeply tied to family, Culture, and Community. As a non‐Indigenous researcher, she remains mindful of the need to listen, learn from, and be guided by Indigenous voices in all aspects of her work*.
**Cliff Whetung**	*Cliff (he/him) is a band member of Curve Lake First Nation, Mississauga Ojibway, Black Duck Clan, and a descendant of English settlers. His work is informed by critical race, decolonization, and health equity theories, and he strives to advance brain health equity in Indigenous communities worldwide*.
**Astrid Suchy‐Dicey**	*Astrid is a non‐Indigenous epidemiologist and researcher, and daughter to scientists in a family of Czechoslovakian and British immigrants and farmers. She was raised throughout different areas of the Southwest, Southern Plains, and Deep South United States. She focused on history and biology in undergraduate studies and later on epidemiology and health disparities in her graduate and post‐graduate work. She has studied risk and resilience in brain aging of American Indian and other Indigenous peoples for the past 15 years, while living in and around the waters, forests, and mountains of Seattle, Los Angeles, and Boston, places that have held her as she reflects and learns. She recognizes her responsibility as a researcher as more than academic, also reflecting a living relationship with Peoples and communities with whom she works. She is committed to the ethical conduct of research that centers Indigenous voices throughout the processes of data gathering and analysis, interpretation, and dissemination of results*.
**Adrienne Withall**	*Adrienne is a Dharawal Yuin and Anglo Celtic woman. She was raised, and is fortunate to still live, surrounded by beautiful Bidjigal lands and waters. Adrienne is a mother and wife, an academic, and an Associate Professor in the School of Psychology at the University of New South Wales. Her research is centered upon amplifying the voice of people aging at the margins, which includes Aboriginal and Torres Strait Islander Peoples, and identifying and addressing equity and social justice issues in dementia research*.
**Kylie Radford**	*Kylie is a woman of Anglo‐Celtic descent, born and raised on Wiradjuri land in rural Australia. Now Sydney‐based, she is a wife and mother, clinical neuropsychologist, and researcher working in partnership with Aboriginal communities across New South Wales to understand and support brain health equity*.
**Diane Carol Gooding**	*Diane is a female scholar of African and Caribbean descent with an ongoing history of allyship with the Indigenous community. Trained as a Ph.D.‐level clinical psychologist, she is part of a research team that uses a community‐based participatory engagement approach in their work with Black and Indigenous communities. Diane is committed to amplifying the voices of underrepresented research groups to reduce health disparities in dementia and to enhance overall health outcomes. As a non‐Indigenous scholar, Diane is grateful to her Indigenous brothers and sisters who teach her through their friendship, cultural practices, writings, and advocacy. As a woman of color, she believes in lifting as she climbs*.
**Louise Lavrencic**	*Louise is a non‐Indigenous woman of European and English heritage. She was born and raised on Kaurna Country, north of Adelaide in Australia, and moved to Sydney after completing her PhD to work as a research academic. Since then, for almost 8 years, Louise has been part of the Aboriginal Health & Ageing Program at Neuroscience Research Australia (NeuRA), committed to working in partnership with Aboriginal communities and listening deeply and respectfully to Elders and community members. During that time, and alongside personal reflection and learning, Louise's commitment to brain health equity has strengthened immensely*.
**Makarena Dudley**	*Makarena is an Indigenous Māori of Aotearoa New Zealand. She belongs to Te Rarawa, Te Aupōuri, and Ngāti Kahu tribes. Makarena trained as a Clinical Psychologist and Clinical Neuropsychologist. She is a Senior Lecturer in the School of Psychology and Deputy Director Māori at the Centre for Brain Research at the University of Auckland. Makarena has been conducting research into dementia and Māori for over a decade. She has led research into the development of the MANA tool for diagnosing dementia in Māori, a dementia app, and the modification of the Cognitive Stimulation Therapy programme for Māori*.
**Dina Lo Giudice**	*Dina is a non‐Aboriginal geriatrician and researcher and first generation Australian of Italian heritage. She is a clinical researcher focusing on the needs of people with dementia, particularly of Aboriginal and Torres Strait Islander people, and those of diverse cultural and linguistic backgrounds for over 20 years*.
**Leon Flicker**	*Leon is a non‐Indigenous clinician researcher born in Sydney whose parents were refugees to Australia from Eastern European and Middle Eastern backgrounds. After working clinically as a geriatrician in Newcastle, Sydney, Melbourne, Alice Springs, Perth, and the Kimberley, he was struck by the impact of the health problems of older Aboriginal and Torres Strait Islander peoples. He is working with Aboriginal and Torres Strait Islander peoples to help address these issues*.
**Arantxa Sanchez Boluarte**	*Arantxa is a physician scientist at the Universidad Peruana Cayetano Heredia. She completed her MPH degree in Global Health at the University of Washington. She was born and raised in Peru. While she is part of the Latinx community, her grandfather was Quechua, the largest Indigenous group in Peru. She leads a brain health study in the Shawi Nation, Indigenous people from the Amazon Rainforest, as part of the NIH Fogarty postdoctoral fellowship in Peru. She is interested in learning about participatory/community‐engaged methods to implement into her study and future proposal*.
**Sulakshna Aggarwal**	*Sukakshna is an ECFMG‐certified MBBS doctor from Maulana Azad Medical College, New Delhi, India. She completed a 6‐month research internship at the Centre for Brain Research, Indian Institute of Science, Bangalore, India. She studied risk and protective factors of dementia in the rural South Indian population from the baseline data of the Centre for Brain Research‐ Srinivaspura Aging, NeuroSenescence, and COGnition (CBR‐SANSCOG) study. She belongs to a non‐Indigenous population of a colonial country that gained independence from British colonial rule in 1947. She deeply believes that the cultural practices and adaptation of the Indigenous people need to be studied and takes immense pride in representing the rural population of India on a global platform*.
**Kyle R Conniff**	*Kyle is a Menominee (Native American) statistician and dementia researcher. His training in survival analysis and methodological development is grounded in a commitment to advancing health equity and Indigenous Data Sovereignty. Raised as the only student of color in a small, predominantly White community in the northern United States, he has experienced both marginalization and the privileges of access to academic opportunity. These experiences shape his responsibility to question how data are collected, analyzed, and interpreted, particularly for American Indian and Alaska Native populations, who have often been misrepresented in health research. He strives for balancing scientific advancement with ensuring Indigenous benefit*.
**Amy Graeme Brodtmann**	*Amy was born on the lands of the Brayakaulung people of the Gunaikurnai nation and has mixed German, Norwegian, British, Indian, and Chinese genetic heritage. She is a professor of neuroscience, a neurologist, and a researcher whose vision and mission are brain health for all. The youngest of three daughters raised by a single mother on Wurundjeri Woi Worrung lands in the outer northeastern suburbs of Naarm/Melbourne and now the mother of three daughters herself, Amy's life experiences support a deep respect for and recognition of holistic approaches to Aboriginal and Torres Strait Islander well‐being*.
**Monica M Diaz**	*Monica is a neurologist at the University of North Carolina at Chapel Hill. She is a non‐Indigenous researcher born in the United State whose parents were political refugees from Cuba. She studies dementia prevalence and risk factors in marginalized populations in low‐to‐middle income settings, including in Peru and Uganda. She co‐leads a study on risk factors for dementia and cognitive impairment among the Shawi, an Indigenous community in the Peruvian Amazon*.
**Stéfanie A Tremblay**	*Stéfanie is a non‐Indigenous early‐career researcher who lives and work in Montreal, Quebec, Canada. She has completed a PhD in neuroimaging, studying the brain structure of older adults at risk of dementia and investigating associations between the so‐called “modifiable risk factors of dementia” and white matter abnormalities in these individuals. Reflecting on the broader implications of her work led her to realize that most interventions aimed at reducing dementia risk perpetuate existing inequities in dementia prevalence and outcomes. This realization led her to pursue postdoctoral work on the social and structural determinants of health. She is leading an initiative aimed at creating a guidance framework for the integration of these determinants in research on aging and dementia in Canada*.
**Emmanuel S Nwofe**	*Emmanuel is an NIHR (DEMCOMM) Fellow focused on applied aging and brain health research within the UK minority ethnic populations. He is originally from the Igbo tribe of southeastern Nigeria. He has been working on dementia prevention, particularly on modifiable risk awareness and reduction within Black African and Caribbean communities in the UK, with a special interest in co‐designing culturally appropriate dementia awareness campaigns that highlight the cultural understanding and practices of people from Black African and Caribbean backgrounds*.
**Carey E Gleason**	*Carey identifies as a White female of Northern European descent. She holds a position as a professor of Geriatrics and Gerontology at the University of Wisconsin*–*Madison, a predominantly non‐Hispanic and White academic institution in the United States. Dr. Gleason acknowledges that she does not have the lived experiences of Black and Indigenous individuals. To enhance her cultural awareness, she seeks out opportunities to learn from courses, seminars, and literature and art. Still, she knows that her identity means that her scientific work will always originate from an outsider's perspective and one of privilege. To address this limitation, she amplifies the voices of those with diverse lived experiences, bringing a range of perspectives to her research work—at all levels. This includes supporting advisory boards, hiring staff who hold marginalized identities, and mentoring the next generation of leaders. She describes herself as the placeholder. She strives to use her time in leadership and privilege to elevate future leaders who represent the communities with whom she partners. Moreover, she wants to make changes to the existing systems, decolonizing institutional policies and practices that limit the inclusion of diverse lived experiences*.
**Kristen Jacklin**	*Kristen is a non‐Indigenous woman from rural northern Ontario, Canada, with Scottish and English roots. She is the Director of the Memory Keepers Medical Discovery Team at the University of Minnesota, a team of scientists deeply committed to research that will preserve the brain health of Elders. She is passionate about supporting Indigenous trainees and building Indigenous dementia research capacity. To this end, she founded the International Indigenous Dementia Network in 2009. She has partnered with First Nations in Canada and Tribal Nations in the United States for the past 25 years. Much of her research has focused on Indigenous dementia, healthy brain aging, and strategies to decolonize biomedicine and Western health care. Her work is guided by community advisory groups and leadership to ensure the research is meaningful and appropriate for diverse Indigenous communities*.
**Joseph Keawe'aimoku Kaholokula**	*Keawe is a Kānaka Maoli (Native Hawaiian) clinical psychologist, health equity researcher, and advocate for the advancement of Native Hawaiian and Pacific Islander health, with direct ancestral ties to Hawaiʻi Island and Maui in the Hawaiian Islands. He is the Chair and a tenured Professor of Native Hawaiian Health at the University of Hawaiʻi's medical school. His primary research focuses on developing culturally grounded, community‐placed health promotion programs to improve the cardiometabolic health of Native Hawaiians and Pacific Islanders. His advocacy in this regard aims to amplify the voices of Indigenous Pacific peoples, unveil their most pressing health concerns, and ensure their inclusion in health research, health care, and public health policies and practices*.
**Chontel Gibson**	*Chontel is a Gamilaraay yinaar, with family connections (from her mother and grandmother) to the Weatherall, Kennedy and Thorne families. Chontel is currently living on Wiradjuri Country. Chontel is able‐bodied, cis‐gender, and uses she/her pronouns. She recently cared for her grandparents while her grandfather was in his final years of living with dementia and other complex health and aging issues. Although Chontel has participated in many Western education programs, she has done so under the guidance and support of Aboriginal and Torres Strait Islander Peoples, both professional and community based. Her professional work sits within the frame of decoloniality, which addresses the ongoing colonial domination that is systemically entrenched in all levels of the Australian society. This decolonial work includes privileging Aboriginal and Torres Strait Islander peoples and social and emotional well‐being in aged care, health, education, and policy*.
**Juliana Souza‐Talarico**	*Juliana, a non‐Indigenous woman of Latin heritage, who was born and raised in Brazil, where she began her research on dementia in underrepresented populations, including Indigenous communities. Since moving to the United States, she has continued to investigate modifiable dementia risk factors, with a focus on psychosocial and environmental stressors and their impact on cognition and dementia risk. Her collaborative work with Indigenous communities in Brazil, combined with her active participation in the International Indigenous Dementia Research Network, has helped amplify Indigenous perspectives and lived experiences in dementia research, promoting culturally informed approaches to prevention and intervention*.
**Pamela Roach**	*Pam is a Métis health systems researcher, originally from the historic Métis community of St. Laurent, Manitoba, Canada. She completed a PhD at the University of Manchester in England and has been working with families living with dementia for close to 20 years. Pam also holds a Canada Research Chair in Indigenous Health Systems Safety to improve the health services available for Indigenous people living with dementia, their families, and their communities with a goal of improving Indigenous health outcomes*.

## 2 CURRENT EPIDEMIOLOGICAL EVIDENCE ON DEMENTIA AMONG INDIGENOUS POPULATIONS

Understanding the burden of dementia among Indigenous peoples requires interrogation of the existing epidemiological evidence and its methodological approach. Although emerging data demonstrate a disproportionately higher prevalence and earlier onset of dementia in many Indigenous populations, these patterns are shaped by the unique socio‐cultural and historical influences on data collection, diagnostic practices, and representation in research.

Although age‐standardized dementia prevalence estimates vary across studies, Indigenous populations consistently demonstrate higher dementia prevalence referent to the dominant national or regional non‐Indigenous population 60 years of age or older in very high Human Development Index (HDI) countries. This pattern has been reported among Aboriginal and Torres Strait Islander peoples in Australia; Māori in Aotearoa New Zealand; First Nations, Métis, and Inuit populations in Canada; and Native Hawaiian and American Indian and Alaska Native populations in the United States.^5^ Similar findings are also seen among Indigenous populations in Asia, including Singaporean Malay and Melanau groups in Malaysia.^5^ Evidence from LMICs suggests a disproportionately high burden of dementia among Indigenous communities, although the picture is more heterogeneous. In the Brazilian Amazon, prevalence among Indigenous groups such as the Mura, Mamirauá, and Amanã, has been reported as more than double the national estimate of 5.8% for older adults.^9^, ^10^, ^14^ Notably, very low prevalence has been observed among the Tsimane and Moseten of Bolivia, whose subsistence‐based agricultural lifestyles and limited exposure to urban risk factors are proposed to offer some protection.^11^, ^14^

The burden of dementia across younger age bands emerges as a consistent finding and may reflect the earlier accumulation of known dementia risk factors within Indigenous populations, as well as the resilient survival of older adults. For example, age‐specific prevalence is up to five times higher for remote‐living Aboriginal and Torres Strait Islander peoples younger than 60 years of age compared to national Australian estimates.^15^ Clinic‐based data show that Native Hawaiians younger than 60 years of age are nearly two times more likely to have dementia compared to non‐Indigenous populations.^16^ Earlier onset of dementia is also frequently reported across most LMIC studies, except for the Tsimane and Moseten, where cases primarily occur after age 80 years.^11^ Younger‐onset dementia confers unique challenges, including disruption of provider roles in family and community systems, thereby compounding financial disadvantage and adding urgency to the need for responsive engagement.^17^

Incidence rates of dementia are less well established among Indigenous populations. The evidence suggests higher incidence among Indigenous peoples relative to non‐Indigenous populations in Australia and the United States.^18^, ^19^, ^20^, ^21^ It is broadly accepted that the number of Indigenous peoples living with dementia is expected to rise due to increasing life expectancy, cohort flow from younger age groups into older age, and strengthened Indigenous identification.^22^, ^23^ Alongside these projected increases, diagnostics point to distinct patterns in dementia subtypes among Indigenous peoples.[Table alz71125-tbl-0001] Although Alzheimer's disease remains the most commonly diagnosed dementia, there is a higher frequency of mixed or unspecified dementia among Indigenous individuals, suggesting the possibility of more pronounced vascular contributions and the need for culturally specific assessment tools.^5^, ^24^ As disease‐modifying therapies gain regulatory approval worldwide, there is a pressing need to consider accurate diagnostic approaches for all peoples at risk of dementia.

Some of the variation seen in dementia epidemiology reflects the diversity of Indigenous experiences: Indigenous cultures, histories, social structures, and health outcomes differ across communities, regions, and contexts. Factors such as geography, social organization, political representation, and access to resources shape both dementia risk and cognitive resilience. Communities with stronger social and political organization may show lower prevalence. Framing prevalence solely at the national level risks masking these distinctions and highlights the need for more community‐level analyses capable of capturing place‐based determinants of dementia.

### 2.1 Refining epidemiological approaches

Longstanding methodological constraints limit robust understanding of dementia among Indigenous peoples. Indigenous populations remain underrepresented in dementia research, often amalgamated with disparate minoritized groups. This approach overlooks the unique positioning of Indigenous peoples as the traditional and rightful owners of lands who bear the impacts of colonization and historical and current policies that influence health equity. Within the context of dementia, Indigenous peoples may engage in differing health care practices, hold unique perspectives on cognitive decline, and rely more heavily on community‐based approaches to care, all of which can delay or obscure medical diagnoses of dementia.^25^, ^26^, ^27^

Administrative datasets, which require a documented diagnosis of dementia in medical records are likely to underestimate case numbers.^28^, ^29^, ^30^ Electronic health records amplify bias and misclassification of Indigenous peoples’ cultural identity, with providers variably categorizing patients and often assuming patients’ race or ethnicity.^31^ Similarly, community‐based epidemiological approaches that rely on standard neuropsychological approaches frequently lack cultural validity in Indigenous contexts. Persistent issues include epistemic racism, culturally inappropriate test items, language barriers, educational bias, and normative data derived from non‐Indigenous populations.^32^, ^33^ For instance, orientation questions such as naming the day or month may be unsuitable in communities where time is understood through environmental or seasonal cues.^10^ Incorporating sociocultural factors such as bilingualism and engagement in cultural practices, often excluded from conventional psychometric frameworks, is vital to understanding cognitive resilience among Indigenous peoples.^34^, ^35^

Several culturally tailored cognitive assessment tools have been developed. The Kimberley Indigenous Cognitive Assessment (KICA) in Australia,^36^, ^37^ the Canadian Indigenous Cognitive Assessment (CICA),^38^ the Māori Assessment of Neuropsychological Abilities (MANA),^39^ the American Indian Cognitive Assessment (AMICA),^40^ the Bharmour adaptation of the Mini‐Mental State Examination (BMSE),^41^ and the Brazilian Indigenous Cognitive Assessment (BRICA)^10^ are adaptations of cognitive assessments developed in partnership with Indigenous communities. These tools incorporate culturally relevant stimuli and account for educational contexts and traditional experiences. More research is needed on their suitability where Indigenous peoples have been moved due to harmful assimilation policies, including off reservations and to urban locations. Although it remains unexplored, the careful development of these tools may have generated cognitive assessments that are more accessible for all peoples, potentially creating opportunities for Indigenous‐centered tools to be the standard, rather than the exception.

Future epidemiological studies must build upon these culturally adapted instruments; they must embed diagnostic approaches within community settings. Such approaches strengthen community‐led diagnostic pathways, enhance local capacity for brain health promotion, and strengthen community–clinician relationships. To capture the impact of dementia more accurately, surveillance strategies should include governance by Indigenous organizations to enable trust and ensure reliable identification of Indigenous peoples, include younger cohorts and develop normative data derived from Indigenous persons.^42^

## 3 STRUCTURAL CONTRIBUTORS TO DEMENTIA RISK AND PREVENTION

Much of the observed variation in dementia epidemiology is attributed to the biomedical risk factors model that neglects the broader contextual determinants that build cognitive reserve and resilience across the life course and shape dementia vulnerability.^43^ Although Indigenous communities globally have made significant strides in asserting sovereign autonomy and reclaiming land rights and cultural heritage, non‐Indigenous systems and structures constrain the capacity of Indigenous peoples to enact meaningful changes to modify dementia risk.^44^, ^45^ We cannot ignore history in shaping the future.

### 3.1 Modifiable dementia risk factors and social determinants of brain health

The 2024 Lancet Commission on dementia prevention and care identified 14 potentially modifiable risk factors spanning early, mid‐, and late‐life stages, representing ≈45% of dementia cases that could theoretically be prevented or delayed through targeted lifestyle interventions.^46^ These lifespan risk factors—educational attainment, hearing loss, vision loss, traumatic brain injury, hypertension, high cholesterol, alcohol consumption, obesity, smoking, depression, social isolation, physical inactivity, diabetes, and air pollution—are fundamentally intertwined with structural determinants and socioeconomic conditions. Emerging research among Indigenous populations reveals both shared risk patterns and distinct community‐specific expressions shaped by historical, cultural, and environmental contexts.^47^, ^48^, ^49^

Educational attainment is generally viewed as a cornerstone of early‐life brain health protection, with each additional year of formal/Western education associated with a reduced risk of dementia.^46^ The inheritance of intergenerational cultural knowledge and practices such as journey‐based experiences, storytelling, medicinal plant knowledge, and craftsmanship is likely to strengthen cognitive resilience among Indigenous peoples.^50^ Yet, most dementia‐related literature and research continue to focus narrowly on the association between reduced years of formal education and elevated odds of dementia, including among Indigenous populations across Australia,^51^ Canada,^52^ Malaysia,^53^ the United States,^54^ Brazil,^9^, ^10^ and Bolivia.^11^

Using years of formal education as the metric, population attributable risk analyses reveal that educational limitations contribute disproportionately to dementia burden among Aboriginal and Torres Strait Islander peoples in Australia and Māori populations in Aotearoa New Zealand compared to respective non‐Indigenous populations,^47^, ^48^ and formal educational attainment is associated with better cognitive health among Indigenous older adults in the United States.^55^ Although culturally adapted cognitive screening tools represent important progress, many continue to reflect aspects of their original Western test design and task structure.^56^ This approach may advantage individuals with some level of formal education, potentially inflating the apparent protective effect of formal schooling. For example, among the Tsimane, an Indigenous Amazonian community in Bolivia, dementia prevalence is extremely low despite minimal formal education.^11^ In contrast, studies in multiethnic urban Indigenous communities in the Brazilian Amazon found no association between education and dementia prevalence, likely reflecting uniformly low education levels and high illiteracy.^9^ Educational attainment may therefore function more as an indicator of socio‐cultural advantage.

These studies highlight the importance of developing epistemologically valid approaches, including Indigenous research methodologies, that recognize local knowledge systems and pathways of cognitive resilience and actively decolonize research practices.^57^ Conventional education metrics may be a poor proxy for baseline cognitive function in many Indigenous contexts, where the quality of schooling has varied considerably due to culturally inappropriate curricula, histories of exclusion from and abuse in educational institutions, and the dispossession of intergenerational cultural knowledge.^58^ The impact of residential or boarding school attendance and the consequent trauma and anxiety when undergoing formal cognitive testing is underappreciated.^59^ Contemporary research suggests that improved access to stimulating employment opportunities, traditional knowledge acquisition, and generative volunteering activities may serve as more culturally relevant proxies for lifelong cognitive stimulation among Indigenous populations.^19^, ^55^, ^60^, ^61^

Employment security, adequate housing, and income stability lay the foundation for brain‐healthy behaviors by mitigating poverty‐related risks and enabling access to protective resources.^62^ Globally, Indigenous households experience poverty rates two to three times higher than national averages, with associated cascading effects on multiple dementia risk pathways.^63^ Economic disadvantage restricts access to nutritious food sources, safe environments for physical activity, and access to preventive health services. Geographic remoteness exacerbates health care accessibility barriers for many Indigenous communities in both HICs and LMICs.^64^ Concurrently, urban migration can reduce intergenerational caregiving networks, amplifying social isolation in older adults.^65^ For example, in a study of the Mura Indigenous Tribe in a remote area of Amazonas, Brazil, completing a full functional assessment for dementia was not possible due to a lack of reliable information from family or close community members, illustrating intergenerational challenges.^9^ These socioeconomic constraints contribute directly to the greater prevalence of established biomedical risk factors during midlife, including increased cardiometabolic disease burden, higher rates of alcohol and tobacco use, vision loss, and untreated hearing loss.^6^, ^66^, ^67^

Indigenous populations residing in LMICs confront additional structural barriers that limit risk‐reduction opportunities, including competing health priorities such as infectious diseases and systemic challenges in implementing prevention strategies.^68^ Resource extraction (oil, mining, logging) and large‐scale agricultural industries in the Amazon and parts of Africa displace communities, erode food sovereignty, and increase exposure to environmental toxins (e.g., heavy metals, pesticides) that directly affect brain health.^69^, ^70^, ^71^ Similar patterns have been documented in North America, where proximity of Indigenous groups to mining operations, industrial waste sites, and contaminated water sources has led to elevated exposures to neurotoxic metals among Indigenous communities.^72^ These LMIC‐specific and cross‐regional exposures necessitate inclusion of an environmental focus during history‐taking and resource‐appropriate prevention frameworks that acknowledge both universal risk factors and region‐specific determinants.

### 3.2 Colonial context and ongoing impacts on brain health

At the root of social and health equity considerations are Indigenous determinants of health.^73^ Colonial policies of dispossession, societal and language assimilation, religious missions, and cultural suppression created systematic disruptions that have direct implications for life course cognitive resilience for Indigenous peoples, with these impacts passed intergenerationally and enduring today.^43^

The forced removal of Indigenous people from ancestral territories represents a profound structural intervention that disrupted place‐based knowledge systems and collective identity, both critical elements of cognitive, social, and emotional well‐being.^74^ Historical and ongoing traumas establish chronic stress pathways beginning in early childhood and contribute to age‐related neuroinflammation and neurodegeneration.^75^, ^76^ Markers of childhood adversity, including separation from family and frequent relocation, have been associated with the development of Alzheimer's disease in Aboriginal and Torres Strait Islander people in Australia.^77^ Lifetime discrimination experiences have also been shown to contribute to lower baseline cognitive scores and progressive cognitive change over time among Indigenous older adults in the United States.^78^ Colonial legacies, although varying across contexts, consistently undermine Indigenous determinants of brain health and cognitive resilience, underscoring the need for systemic transformations at all levels of society. Emerging research highlights the importance of cultural and political determinants of health, including the ways Indigenous peoples assert self‐determination, as key to health and well‐being, including brain health.

## 4 INTEGRATING INDIGENOUS PERSPECTIVES INTO BRAIN HEALTH AND DEMENTIA CARE

For many Indigenous communities, cognitive changes in later life reflect holistic frameworks. As such, protective factors for brain health and dementia caregiving are often framed collectively, challenging Western biomedical frameworks and government‐funded systems of support that focus on the individual.

### 4.1 Indigenous brain health in the context of cognition, ageing, and dementia

There is growing recognition of cultural approaches to cognitive health and aging among Indigenous populations.^26^, ^32^, ^79^, ^80^, ^81^, ^82^ In some Indigenous cultures, these changes are interpreted as a form of transition that draws individuals closer to ancestral knowledge and spiritual continuity.^83^, ^84^, ^85^, ^86^ Accordingly, dementia may be accepted in Indigenous communities, with family and community members emphasizing inclusion of older Māori in cultural activities, for example.^32^ In some settings, preservation of brain health is approached collectively, reinforcing intergenerational responsibilities and sustaining cultural knowledge transmission.^27^ However, limited awareness of the pathological underpinnings of dementia, compounded by negative past experiences with health care systems, may contribute to underdiagnosis and feelings of shame and stigma surrounding cognitive decline.^26^, ^80^, ^87^ These dynamics are further complicated by the marginalization of Indigenous perspectives within health care services, which often fail to engage meaningfully with local cultural and linguistic frameworks.^32^, ^43^ Optimizing dementia prevention and care requires the development of educational and health policy approaches that sustain cognitive health for the collective.

### 4.2 Support for culturally protective approaches to brain health

Acknowledging the centrality of culture, kinship, and connection to land and community broadens perspectives on brain health beyond biomedical framings.^88^ A decisive move away from deficit‐based narratives also illuminates how cultural assets and inherent resilience within Indigenous communities can be leveraged as powerful neuroprotective resources.^89^

Indigenous perspectives and lived experience accentuate the relationship between social connection, holistic concepts of well‐being, and cognitive resilience.^90^ Evidence already demonstrates that social isolation is a major risk factor for neurodegeneration.^46^ Research among Māori peoples in Aotearoa New Zealand and Aboriginal and Torres Strait Islander peoples in Australia reveals significantly lower rates of social isolation than respective non‐Indigenous populations, reflecting the foundational importance of *whānau* (family) and community structures.^47^, ^48^ Strengthening these elements through cultural participation and intergenerational engagement challenges Western biomedical frameworks, which frequently isolate dementia as a disease of the individual.^83^, ^87^

Other avenues for promoting brain health among Indigenous populations, grounded in traditional knowledge and practices such as dance, language, and social connection show promise in reducing dementia risk globally. For example, in Brazil, federal initiatives arising from sustained Indigenous advocacy promote Indigenous languages and cultures through the adoption of bilingual and intercultural curricula and Indigenous teacher training.^91^ In the United States, the Strong Heart Study is collating evidence on how self‐perception, identity, and cultural factors influence cognitive health among American Indian adults.^34^ In Hawai'i, the *Kā‐HOLO* study used *hula*, a traditional Native Hawaiian dance and cultural practice, as the foundation for a culturally grounded lifestyle intervention that significantly improved hypertension, self‐care, and cardiovascular risk.^92^ This approach has since been adapted to target vascular and metabolic risk factors for dementia among Native Hawaiians and Pacific Islanders as part of the *‘IKE Kupuna* (Elder Wisdom) project.^93^ Highlighting the importance of involving young people in brain health approaches, the *Hā Kūpuna* National Resource Center for Native Hawaiian Elders also developed a community‐informed dementia educational storybook for Native Hawaiian youth.^94^ In Australia, the Sharing the Wisdom of our Elders project enhances dementia prevention knowledge through cultural expression and intergenerational learning,^95^ while the first randomized controlled trials examining community‐led approaches to reduce dementia risk in Aboriginal communities are also underway.^96^, ^97^ Taiwan's “Dementia Classrooms” and “Intergenerational Learning Programs” integrate Indigenous language education with Elder‐led cultural activities, preserving cultural heritage while supporting cognitive health.^98^ Indigenous‐led innovations like these demonstrate culturally grounded pathways to dementia prevention and underscore the leadership of Indigenous communities in advancing global brain health equity.

### 4.3 Quality and cultural propriety of dementia care

Within Indigenous communities, caregiving often occurs collectively, with decision‐making shared across family or kinship networks.^32^ These relational approaches challenge Western egocentric norms in health care, such as individual consent and autonomy, which may be culturally incongruent.^27^ Indigenous‐led, strengths‐based models of care offer clinicians, policymakers, and researchers pragmatic strategies to engage respectfully and collaboratively with Indigenous peoples.^99^, ^100^ Such models prioritize social and cultural reconnection, for example, enabling Elders to age *in* place,^101^ rather than defaulting to institutionalization or exclusively medical management.^102^, ^103^

Across the world, Indigenous‐led and collaborative initiatives are transforming dementia care. In Canada, the Peter Ballantyne Cree Nation's Integrated Care Model offers valuable lessons in shared caregiving for First Nations Elders,^104^ whereas in the United States, research collaborations such as the Northwest Tribal Elders Project^105^ and the *‘Auamo Kuleana O Nā Maʻi Poina* in Hawai'i^106^ provide important models for culturally appropriate training and resource development for caregivers and providers. The longstanding Strong Heart Study exemplifies how enduring partnerships between American Indian communities and research institutions can produce both scientific insight and tangible community benefit.^107^ In Australia, the Let's CHAT (Community Health Approaches To) Dementia program represents a significant national initiative that co‐designed models of care within Aboriginal and Torres Strait Islander health services, demonstrating early promise in improving dementia diagnosis and management.^108^, ^109^ The partnership between *Te Hau Ora o Ngāpuhi* and the University of Auckland in Aotearoa New Zealand demonstrates meaningful integration of Māori cultural values and practices into dementia care,^110^ while partnerships in Manaus, Brazil, are informing national dementia policy through community‐based research.^111^

These initiatives show that culturally responsive dementia care is achievable when it is based on genuine partnership between Indigenous communities and research institutions. Yet they also underscore the sustained time, resources, and trust required to build and maintain such collaborations, challenges often at odds with short‐term funding cycles. Ensuring sustainability demands evaluation frameworks that pair Indigenous‐defined measures of success, such as community well‐being, cultural continuity, and family resilience, alongside conventional biomedical or clinical outcomes.

### 4.4 Integrating Indigenous approaches alongside biological advances

It is equally critical that Indigenous peoples are included in advances in understanding the genetic and biomarker signatures of dementia alongside culturally aligned approaches to prevention and care. Inclusion enables identification of both shared and unique risk and protective factors for cognitive ageing. Currently, the relationship between biomarkers and ethnoracial background remains poorly studied. A recent review found that investigations of Alzheimer's disease biomarkers in Indigenous groups is scarce, with most work focused on the apolipoprotein E (*APOE*) ε4 allele.^6^ Findings were inconsistent with half of the identified studies (*n* = 12) reporting no association between *APOE* ε4 and dementia and wide variation in allele prevalence (4.6%–24%) among Indigenous populations.^112^ Evidence from neuroimaging is similarly limited, with no positron emission tomography (PET) imaging or cerebrospinal fluid (CSF) biomarker research to date.

The lack of robust biomarker data underscores the need for more inclusive research approaches. Blood‐based biomarkers may be particularly valuable for their accessibility and cultural acceptability.^113^ However, biomarker thresholds must be validated in Indigenous populations rather than relying on cut‐points derived from predominantly non‐Hispanic White samples. The genetics of Alzheimer's disease in peruvian populations study in Peru, which included participants with an average of 80% Native American ancestry demonstrated that plasma phosphorylated tau‐217 (p‐tau217) was significantly associated with Alzheimer's disease, an important extension of the literature in this field.^114^ In the United States, the Strong Heart Study examined plasma markers in older American Indian adults and found significantly lower amyloid (amyloid beta [Aβ]42 and Aβ42/40) levels compared to non‐Hispanic White and other groups, suggesting a distinct trajectory of Alzheimer's disease pathology.^115^ The authors propose that American Indian individuals may experience earlier or accelerated amyloid accumulation, further highlighting that current dementia risk estimates may be understated.

Although the promise of biomarkers is clear, their application must be guided by robust ethical responsibility that ensure Indigenous research governance and adherence to relevant Indigenous ethics protocols. Research benefits must extend beyond typical Western scientific outputs to include tangible investments that reflect Indigenous peoples’ aspirations. These benefits may include culturally safe services to address prevention of brain health issues, health, education, and training of future Indigenous clinicians and researchers.

### 4.5 Advancing Indigenous leadership in dementia research and management

Many Indigenous communities are justifiably skeptical of health researchers given overwhelming histories and continuation of abuse and exploitation.^116^, ^117^ Despite this, older Indigenous peoples and advocates consistently express interest in collaborative research partnerships.^83^, ^118^, ^119^, ^120^ A new generation of Indigenous scientists is also growing in power within the academy, and is actively engaged in mentorship of the next generation.^121^, ^122^ In the United States, more than 12,000 American Indian and Alaska Native individuals have been awarded doctoral degrees since 2007, with similar trends emerging globally. These scholars are leading efforts to interrogate, decolonize, and embed Indigenous research methods. For example, one approach to advancing Indigenous health centers on analyzing unused data from Indigenous research participants whose contributions to science are often erased by limited efforts to disaggregate “other” racial and ethnic categories in longitudinal data.^123^

Modern statistical approaches allow meaningful inferences from smaller sample sizes, enabling more granular, population‐specific analyses. Yet researchers can be deterred by stringent data‐protection measures that slow research pipelines and impose strict security and reporting protocols.^124^ Informed by data sovereignty principles of ownership and access, these safeguards are essential for ensuring participant protection but can inadvertently limit Indigenous representation in dementia research. Indigenous scientists, however, are increasingly leading this work, supported by networks of Indigenous and non‐Indigenous mentors experienced in navigating these challenges. For example, the International Indigenous Dementia Research Network, founded in 2009, exemplifies such collaboration, bringing together over 200 scholars from Australia, Aotearoa‐New Zealand, Brazil, Canada, and the United States in 2024 to share scientific advances and mentorship. Initiatives like this strengthen Indigenous leadership in policy, research, and intervention development while fostering the next generation of Indigenous scholars.

### 4.6 Research and health policy implications

**FIGURE 2:**
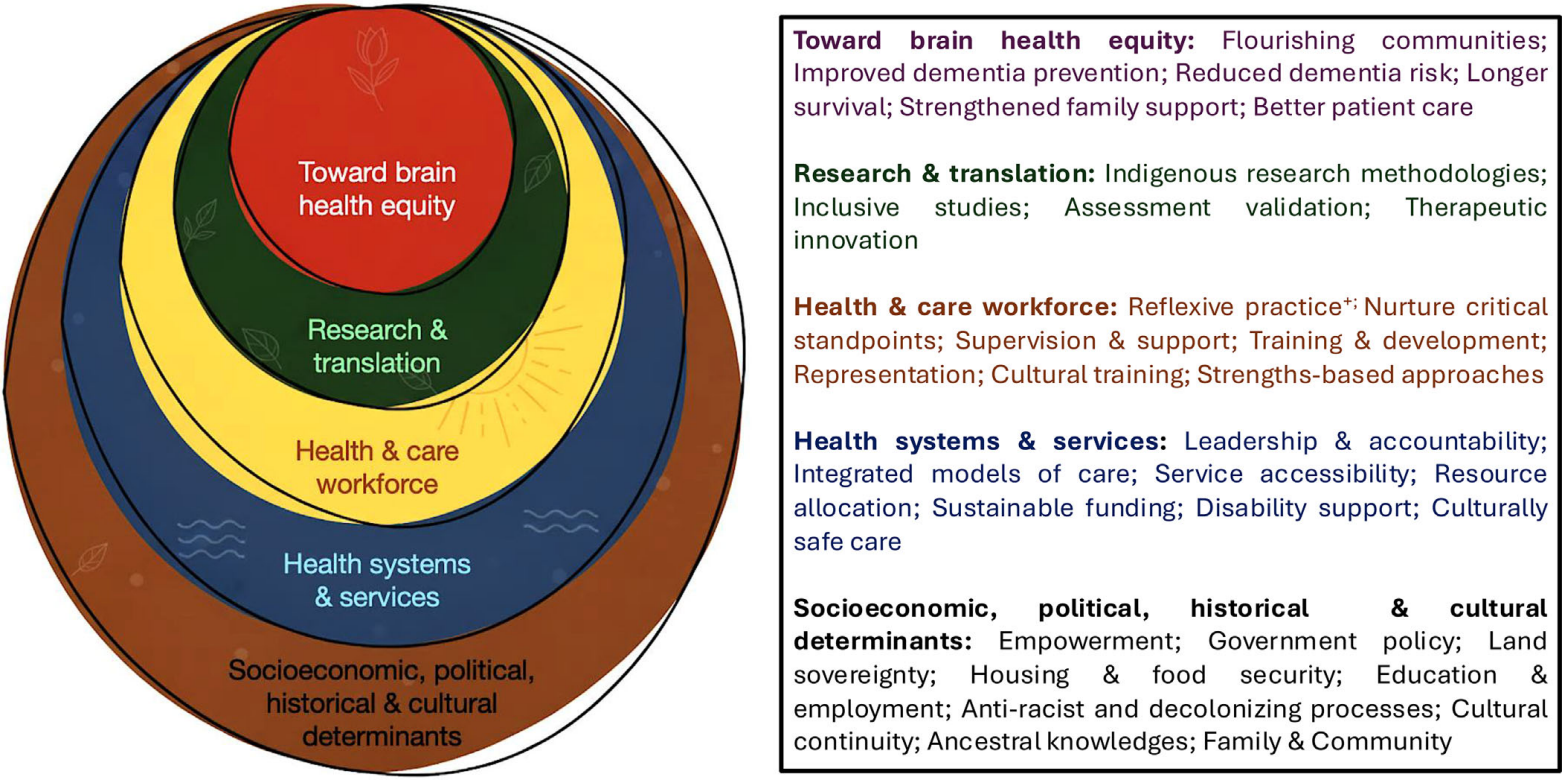
Achieving brain health equity with Indigenous peoples requires addressing the intersecting layers that shape cognitive resilience. These include structural determinants, environmental influences, health systems and service design, the quality of clinical, aged and disability care, and active engagement in decolonizing research and clinical practices. ^+^The process of examining one's own cultural identity, biases, positioning and power, and reflecting on the potential influence on relationships.^128^

Developing Indigenous‐specific approaches to dementia prevention and care requires a paradigm shift in both research and health policy. Traditional biomedical models often fail to account for the political, sociocultural, and structural determinants that shape cognitive resilience in Indigenous communities. Rather than frame these approaches as “racialization” of dementia research,^125^ we advocate for the recognition and embedding of cultural frameworks, relational worldviews, and community engagement within health systems, research design, and publication standards (Figure 2).^126^, ^127^, ^128^ Strengths‐based community‐led models that prioritize cultural connection, community knowledge, and environmental stewardship have the potential to build cognitive reserve, and are highly relevant for everyone, yet remain underrepresented in national dementia strategies.^129^, ^130^

Collecting data on structural and sociocultural determinants of health in Alzheimer's Disease Research Centers is a crucial step toward addressing inequities in dementia research.^131^ Integrating these approaches to knowledge creation will advance our understanding of population‐level dementia risks and improve our ability to quantify resilience against dementia. With enough advocacy, such approaches may create the opportunity to include data on the Indigenous determinants of health. The findings of the Lancet commission, alongside the broader literature of lifespan risk and protective factors for dementia, make it clear that the origins of dementia inequities are structural.^49^ They are also historical. Without robust frameworks for capturing the influence of systemic racism, colonial dispossession, and environmental injustice on cognitive health, health policies risk reinforcing existing disparities.

Effective implementation of preventative policies requires integrating Indigenous governance structures into policy development and respecting community protocols around data sovereignty.^132^ Research partnerships must be built on long‐term relationships, capacity strengthening, and co‐design principles that involve community leadership from the outset. Investment in culturally adapted diagnostic tools and education materials can enhance early detection and reduce stigma.^10^ Health workforce development is also critical, including training Indigenous clinicians and health scientists, and embedding cultural safety practices across all levels of dementia care and research.^133^

More broadly, holistic, community‐anchored, and strengths‐based strategies—long practiced in Indigenous contexts—point to preventive pathways that emphasize connection, identity, emotional well‐being, and the structural conditions that enable people to thrive. While any cross‐population application must remain grounded in Indigenous sovereignty, articulating how these principles may inform more responsive models of dementia risk reduction at the community level presents an important conceptual contribution to this field. Future work should therefore continue to prioritize Indigenous leadership while exploring the wider relevance of these approaches to reimagined brain health globally.

## 5 KEY RECOMMENDATIONS

It is not uncommon for well‐intentioned researchers and clinicians to seek guidance on how to “help” Indigenous communities. A central aim of this article is to demonstrate that Indigenous communities and their allies are already leading efforts to preserve brain health globally. What is needed now is genuine partnership. The pain that dementia inflicts on Indigenous communities is magnified by the disproportionate loss that occurs when the memories of Elders fade—each life story represents an irreplaceable link to collective histories, languages, and cultural knowledge. The urgency to prevent and treat dementias stems not only from a commitment to health equity, but from the existential threat these diseases pose to cultural continuity. The challenge before all scholars and practitioners is to act as respectful partners: to listen, learn, and support community‐driven solutions in the pursuit of brain health equity.

- Invest in Indigenous‐led brain health initiatives. Direct flexible funding to Indigenous‐controlled health services and Indigenous‐led research teams to develop culturally appropriate cognitive assessment, support programs, and dementia prevention initiatives.
- Address structural determinants of health through intersectoral approaches. Brain health equity requires policies that address Indigenous peoples’ sovereignty, education, housing, food security, environmental protection, and economic opportunity.
- Broaden health prevention programs, including evidence‐based science, to optimize cognitive resilience. Support for cultural continuity, language revitalization, access to traditional foods, intergenerational knowledge transfer, and ceremonial participation represent novel approaches to prevention aligned with Indigenous values.
- Include Indigenous peoples as researchers, leaders, stewards, and participants in clinical trials and cohort studies to develop appropriate normative data, informed by Indigenous determinants of health and data sovereignty principles.
- Implement culturally safe, trauma‐informed, and strengths‐based dementia care models that privilege Indigenous healing practices, honor Indigenous human rights, respect cultural protocols surrounding aging and end‐of‐life care, and are supported by sustainable, long‐term funding.
- Build the clinical and research workforce capacity. Increase pathways for Indigenous participation in health professions, sciences and leadership through scholarships, mentorship programs, and curriculum reform in health professional and health sciences education. Support non‐Indigenous clinicians and researchers to be critically reflective and engage in decolonizing practices.

APPENDIX: COLLABORATORS

## AUTHOR CONTRIBUTIONS

**Antonia J Clarke**: Conceptualization; visualization; writing—original draft; writing—review and editing. **Cliff Whetung**: Conceptualization; writing—original draft; writing—review and editing. **Astrid Suchy‐Dicey**: Conceptualization; visualization; writing—review and editing. **Adrienne Withall**: Visualization; writing—review and editing. **Kylie Radford**: Conceptualization; visualization; writing—review and editing. **Diane C Gooding**: Conceptualization; writing—review and editing. **Louise Lavrencic**: Writing—review and editing. **Makarena Dudley**: Conceptualization; writing—review and editing. **Dina Lo Giudice**: Writing—review and editing. **Leon Flicker**: Writing—review and editing. **Arantxa Sanchez Boluarte**: Conceptualization; writing—review and editing. **Sulakshna Aggarwal**: Conceptualization; writing—review and editing. **Kyle Conniff**: Conceptualization; writing—review and editing. **Amy G Brodtmann**: Conceptualization; writing—review and editing. **Monica M Diaz**: Conceptualization; writing—review and editing. **Stéfanie Tremblay**: Conceptualization; writing—review and editing. **Emmanuel S Nwofe**: Conceptualization; writing—review and editing. **Carey E Gleason**: Conceptualization; writing—review and editing. **Kristen Jacklin**: Writing—review and editing. **Joseph Keawe'aimoku Kaholokula**: Writing—review and editing. **Chontel Gibson**: Conceptualization; writing—original draft; writing—review and editing. **Juliana Souza‐Talarico**: Conceptualization; writing—original draft; writing—review and editing. **Pamela Roach**: Conceptualization; writing—original draft; writing—review and editing.

## CONFLICT OF INTEREST STATEMENT

The authors declare no conflicts of interest arising from the research, authorship and/or publication of this article. Any author disclosures are available in the Supporting Information.

## Supporting information



Supporting Information
